# Shortcutting the diagnostic odyssey: the multidisciplinary Program for Undiagnosed Rare Diseases in adults (UD-PrOZA)

**DOI:** 10.1186/s13023-022-02365-y

**Published:** 2022-05-23

**Authors:** Nika Schuermans, Dimitri Hemelsoet, Wim Terryn, Sanne Steyaert, Rudy Van Coster, Paul J. Coucke, Wouter Steyaert, Bert Callewaert, Elke Bogaert, Patrick Verloo, Arnaud V. Vanlander, Elke Debackere, Jody Ghijsels, Pontus LeBlanc, Hannah Verdin, Leslie Naesens, Filomeen Haerynck, Steven Callens, Bart Dermaut, Bruce Poppe, for UD-PrOZA, Jan De Bleecker, Patrick Santens, Paul Boon, Guy Laureys, Tessa Kerre

**Affiliations:** 1 Center for Medical Genetics, Ghent University Hospital, Ghent, Belgium; 2 Department of Biomolecular Medicine, Faculty of Medicine and Health Sciences, Ghent University, Ghent, Belgium; 3 Department of Neurology, Ghent University Hospital, Ghent, Belgium; 4 Department of Nephrology, Jan Yperman Hospital, Ieper, Belgium; 5 Department of General Internal Medicine, Ghent University Hospital, Ghent, Belgium; 6 Department of Pediatrics, Division of Pediatric Neurology and Metabolic Diseases, Ghent University Hospital, Ghent, Belgium; 7 Department of Human Genetics, Radboud Institute for Molecular Life Sciences, Radboud University Medical Center, Nijmegen, The Netherlands; 8 Department of Internal Medicine and Pediatrics, Ghent University Hospital, Ghent, Belgium; 9 Primary Immunodeficiency Research Lab, Center for Primary Immunodeficiency Ghent, Jeffrey Modell Diagnosis and Research Center, Ghent University Hospital, Ghent, Belgium

**Keywords:** Rare diseases, UD-PrOZA, Diagnostic odyssey, Whole exome sequencing, Diagnostic yield, *MAP3K7*, *SGO1*, *SNORD118*, *IRF2BPL*, *PLAAT3*, *ACMSD*

## Abstract

**Background:**

In order to facilitate the diagnostic process for adult patients suffering from a rare disease, the Undiagnosed Disease Program (UD-PrOZA) was founded in 2015 at the Ghent University Hospital in Belgium. In this study we report the five-year results of our multidisciplinary approach in rare disease diagnostics.

**Methods:**

Patients referred by a healthcare provider, in which an underlying rare disease is likely, qualify for a UD-PrOZA evaluation. UD-PrOZA uses a multidisciplinary clinical approach combined with state-of-the-art genomic technologies in close collaboration with research facilities to diagnose patients.

**Results:**

Between 2015 and 2020, 692 patients (94% adults) were referred of which 329 (48%) were accepted for evaluation. In 18% (60 of 329) of the cases a definite diagnosis was made. 88% (53 of 60) of the established diagnoses had a genetic origin. 65% (39 of 60) of the genetic diagnoses were made through whole exome sequencing (WES). The mean time interval between symptom-onset and diagnosis was 19 years. Key observations included novel genotype–phenotype correlations, new variants in known disease genes and the identification of three new disease genes. In 13% (7 of 53), identifying the molecular cause was associated with therapeutic recommendations and in 88% (53 of 60), gene specific genetic counseling was made possible. Actionable secondary findings were reported in 7% (12 of 177) of the patients in which WES was performed.

**Conclusion:**

UD-PrOZA offers an innovative interdisciplinary platform to diagnose rare diseases in adults with previously unexplained medical problems and to facilitate translational research.

**Supplementary Information:**

The online version contains supplementary material available at 10.1186/s13023-022-02365-y.

## Background

In Europe rare diseases are defined as diseases with a prevalence of less than 5/10.000, in the United States diseases that affect less than 200.000 Americans are classified as rare [1–3]. To date, over 6000 rare diseases have been identified, affecting 3.5–5.9% of the worldwide population [4]. Rare diseases are often chronic and disabling and therefore represent a major public health challenge. The majority of rare diseases (80%) have a genetic origin [1]. The rarity complicates research in this field due to recruitment issues on the one hand, and a lack of interest of investors and pharmaceutical companies on the other [5]. In 2010 the Directorate-General for Research and Innovation of the European Commission (EC), together with the US National Institutes of Health (NIH), founded the International Rare Disease Research Consortium (IRDiRC), intended to establish a global partnership to advance rare disease research [3]. International collaboration via secure data exchange platforms, e.g. Matchmaker Exchange [6, 7] , together with the advent of next-generation sequencing (NGS) technology, enabling hypothesis-free approaches, have boosted the discovery of new disease genes over the past decade [8]. The diagnostic yield for whole exome sequencing (WES) and whole genome sequencing (WGS) in the literature ranges from 3 to 70% depending on the patient cohort analyzed [9]. An early implementation of WES in pediatric cohorts presenting with neurodevelopmental disorders has proven to be cost-efficient [10]. Moreover, NGS enables the identification of more than one disease-causing variant in one patient. In a large NGS cohort, approximately 5% of the patients were diagnosed based on pathogenic variants in two or more disease loci [2]. Despite this tremendous progress in rare disease knowledge, 50% of the patients with the suspicion of a rare genetic disease remain undiagnosed [11]. Techniques to address this missing heritability (WGS, epigenomics, transcriptomics) are becoming increasingly incorporated in diagnostics, putting emphasis on the importance of regulatory and non-coding sequences [2]. To elaborate on potential novel disease genes or rare sequencing variants of unknown clinical significance (VUS), in vivo and in vitro functional assays are indispensable [7, 8, 12, 13]. As such, boundaries between diagnostics and research are becoming increasingly blurred, and close collaboration between clinicians, geneticists, molecular biologists, model organism experts and bioinformaticians is necessary. The identification of the molecular cause of a rare disease not only has important psychological consequences for patients and their families, in some cases (approximately 20%) it can lead to changes in recommendations regarding therapy [14]. Pinpointing the underlying genetic cause of a rare disease or identifying a genetic vulnerability predisposing for more frequent disorders, are prerequisites ultimately paving the way for precision medicine. Even in cases where the identification of the molecular cause has no therapeutic implications, it provides important prognostic information and enables correct genetic counseling regarding recurrence risk, predictive testing in relatives, prenatal genetic testing and pre-implantation genetic diagnosis.

Rare disease initiatives all over the world are being established and have reported impressive diagnostic yields ranging from 35–67%, including predominantly pediatric patients with undiagnosed diseases [14–19]. Data on the efficiency of comparable programs in adult patient cohorts with unsolved medical problems are lacking.

In 2015, UD-PrOZA (Program for Undiagnosed Rare Diseases or ‘Programma voor Ongediagnosticeerde Zeldzame Aandoeningen’ in Dutch) was founded at the Ghent University Hospital. Four clinicians with different areas of expertise (genetics, neurology, infectiology/immunology, nephrology) created a multidisciplinary platform to deal with diagnostically challenging cases. Shortly after, the team was reinforced by a neuro-geneticist, bioinformatician, molecular geneticist, clinical genetics trainee and a pediatrician. The primary aim of the initiative is to facilitate the diagnostic process for adult patients suffering from an undiagnosed rare disease. In 2019, UD-PrOZA became part of the Undiagnosed Disease Network International (UDNI) and it got involved in ‘Solve-RD’, a rare disease research project funded by the EC for five years (2018–2022).

## Methods

In this study we report the establishment and the five-year diagnostic results of our multidisciplinary platform aiming to diagnose predominantly adult patients with rare diseases, UD-PrOZA. We evaluated all referred cases between July 2015, until June 2020. Patient characteristics, main clinical features, type of referral and diagnostic yield were determined. Data were gathered from a UD-PrOZA registry. Statistical analysis was performed in SPSS 26 (IBM Corp. Released 2019. IBM SPSS for Windows, Version 26.0. Armonk, NY: IBM Corp). The study was approved by the Ethics Committee of the University Hospital of Ghent (EC: 2019/1430). Written informed consents were obtained from all patients included in this study in which exome sequencing and/or multi-omics analyses were performed.

Patients with unsolved medical problems can be referred to UD-PrOZA by a healthcare provider. The medical records of all applicants are evaluated by a multidisciplinary team that decides on the likelihood of an underlying rare disease. Prior evaluation in routine diagnostic setting and the presence of at least one objectifiable disease sign are prerequisites for acceptance. If a rare disease is suspected, patients are invited for further evaluation after which in most cases, genetic testing is initiated. UD-PrOZA uses multi-omics strategies, model organisms (*Drosophila* and zebrafish facilities) and data sharing platforms to elaborate on novel disease variants or potential novel disease genes. An overview of the UD-PrOZA workflow is provided in figure S1.

## Results

### Study population

From July 2015, through June 2020, a total of 692 patients were referred to UD-PrOZA (Figure S2A). The patient characteristics are summarized in Table 1. 59% of the referrals were female, 94% were 18 years of age or older. The mean age of the patients was 42 ± 16 years. 46% of the cases were referred by their general practitioner, 37% were referred by a specialist working at the Ghent University Hospital. Neurological involvement was reported in 42% of the applicants. In respectively 22% and 18% of the patients, the symptoms were related to autoimmunity/immunology and the musculoskeletal system (Figure S2B). Out of 692 referrals, 329 patients (48%) were accepted for further UD-PrOZA evaluation and follow-up. Only patients in which routine diagnostics have failed to render a diagnosis and patients presenting with at least one objectifiable disease sign were eligible for acceptance. The likelihood of acceptance depended significantly on the nature of the presenting symptoms and the referring physician. Patients with multiple objectifiable disease signs or symptoms were 14 times more likely to be accepted (OR 14.1; 95% CI 9.5–21.0, p < 0.0001). Patients referred by a specialist were 8 times more likely to be accepted compared to patients referred by their general practitioner (OR 8.5; 95% CI 5.9–12.2, p < 0.0001). In seven patients a clinical diagnosis was made after a multidisciplinary consultation (Additional file 1: Figure S2E, Table S1, Table 2). In 237 out of 329 accepted patients (72%) genetic testing was initiated. The remaining 85 patients were referred to another trajectory since the clinical presentation in these cases was not compatible with an underlying rare disease. Within the group of patients in which we requested genetic testing, 59% (139 out of 237) underwent previous genetic testing consisting of targeted genetic tests (89%) and WES-based testing (26%). In 53 patients a definite molecular cause was identified after genetic testing, in 30 patients a potential causal variant was found (functional or segregation data are required for further classification). This results in a total diagnostic yield of 18% in patients with completed workup. Within the group of patients in which genetic testing was initiated, 22% received a definite molecular diagnosis. Remarkably, all diagnosed patients initially presented with objectifiable disease signs. Secondary findings were reported in 7% of the patients in which WES was performed (Table 2, Additional file 1: Table S2).

**Table 1:** Patient information of all referrals, of the accepted referrals and of the patients that have been diagnosed by UD-PrOZA

	All referrals (n = 692)	Accepted referrals (n = 329)	Diagnosed patients (n = 60)
Mean age (years ± SD)	42 ± 16	40.5 ± 16	39 ± 14
Sex (%)			
Male	277 (41)	144 (44)	30 (50)
Female	400 (59)	183 (56)	30 (50)
Referred by (%)			
General practitioner	292 (46)	67 (21)	7 (12)
Specialist	348 (54)	249 (79)	51 (88)
Complaint (%)			
Objectifiable	386 (60)	285 (87)	60 (100)
Not objectifiable	259 (40)	44 (13)	0 (0)
Primary symptoms (%)			
Neurologic	270 (42)	177 (54)	35 (58)
Immunologic/infectious	133 (21)	63 (19)	8 (14)
Musculoskeletal	107 (17)	20 (6)	2 (3)
Rheumatologic	25 (4)	15 (5)	0 (0)
Cardiac/vascular	19 (3)	13 (4)	2 (3)
Gastrointestinal	18 (3)	5 (2)	1 (2)
Other	74 (11)	36 (11)	12 (20)

**Table 2:**
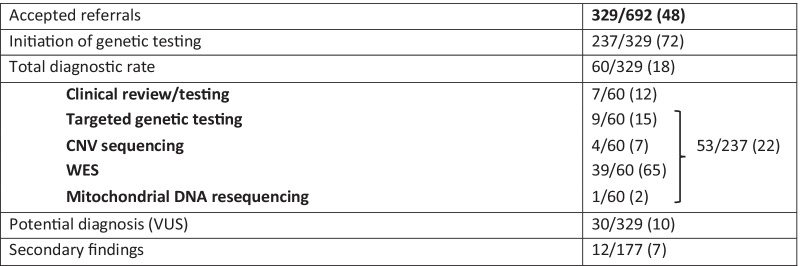
Overview of accepted cases, diagnosed cases and cases with secondary findings; absolute and relative (%)

### 52 genetic diagnoses after a diagnostic odyssey of 19 year on average

88% (53/60) of the established diagnoses had a genetic origin. In Table S3 an overview of all genetic diagnoses is provided. Though all patients were adults at the time of diagnosis, some patients presented the first symptoms at an early age, emphasizing the challenging diagnostics in the field of rare diseases. Strikingly, more than half of the diagnosed patients (56%) had a negative family history: in 19% (10/53) a de novo pathogenic variant was found associated with an autosomal dominant disorder, 26% (14/53) were diagnosed with an autosomal recessive disorder, 6% (3/53) were diagnosed with an X-linked recessive disorder, in 2% (1/53) a pathogenic mosaic variant was identified and in 2% (1/53) a pathogenic mitochondrial DNA (mtDNA) variant in 60% heteroplasmy was found (Figure S2C, Table S3). In the other half of the patients (44%), a positive family history was seen: 25% (13/53) inherited the pathogenic variant from an affected parent, in 17% (9/53) no parental segregation analysis was performed but the family history was positive making inheritance highly likely (Additional file 1: Table S3). Consanguinity was reported in 11 cases. In 27% (3/11) homozygous pathogenic variants were detected (Additional file 1: Table S3). In total, 63 disease causing variants were detected, 27 of which (43%) have never been reported in Clinvar or in the literature to our knowledge. All variants were classified as (likely) pathogenic based on the ACMG classification criteria [20] provided in Table S3. Causal variants comprised missense variants (46%), frameshift variants (14%), nonsense variants (11%), non-coding variants (11%), larger insertion-deletion variants (10%), splice site variants (6%) and mitochondrial DNA variants (2%) (Additional file 1: Figure S2D, Table S3). The majority of the genetic diagnoses were established after WES (65%), followed by targeted genetic testing (15%) and CNV sequencing (7%) (Table 2, Additional file 1: Figure S2E). Of the cases that were diagnosed through WES, 79% were analyzed as singleton, whereas 21% were analyzed as duo or trio with affected and/or unaffected family members (Additional file 2: Table S3). For the patients with a confirmed molecular diagnosis, the time interval between symptom-onset and the moment of diagnosis was estimated at an average of 19 years, ranging from 1 to 52 years (Additional file 2: Table S3). Interestingly, three patients were molecularly diagnosed with more than one rare disease. Our diagnostic strategy had a major impact on the patient level by ending the diagnostic quest and by providing therapeutic options in some cases, but also on a scientific level, including expansion of the phenotypic spectrum and the discovery of three new monogenic diseases.

#### Patients diagnosed with multiple rare diseases

In three patients (Additional file 2: Table S3: ID 14, 19, 26) we identified pathogenic variants in more than one disease gene.

Patient 19, a man with a history of liposarcoma, prostate carcinoma and multiple neurofibromas was referred to UD-PrOZA because of rapidly progressive intracerebral white matter lesions resulting in a state of stupor and eventually coma. Biopsy of brain tissue was indicative of a low-grade glioma. WES revealed heterozygous likely pathogenic missense variants in *NF1* (p.Leu2323Pro) (Neurofibromatosis type 1, MIM: 162200) and in *TP53* (p.Arg282Gln) (Li-Fraumeni syndrome, MIM: 151623) (Additional file 2: Table S3). The *NF1* variant was presumably inherited from his mother, who was also diagnosed with neurofibromas. No segregation analysis in the parents could be performed. Pathogenic variants in both *NF1* and *TP53* have been associated with malignant CNS tumors.

Patient 14 presented with polycystic kidney disease and progressive leukoencephalopathy. Prior targeted genetic testing detected a pathogenic variant in *PKD1* (p.Tyr1159Ter) (Polycystic kidney disease 1, MIM: 173900). WES identified a missense variant in *COL4A1* (p.Gly382Ser) (Microangiopathy and leukoencephalopathy, MIM: 618564) with pathogenic in silico predictions. The variant segregated with the disease in the family and was therefore classified as likely pathogenic.

In patient 26, a female presenting with polycystic kidney disease and noncompaction cardiomyopathy, WES revealed pathogenic variants in *PKD1* (p.Cys3081Arg) (Polycystic kidney disease 1, MIM: 173900) and *TTN* (p.Trp16598Ter) (Cardiomyopathy, dilated, 1G, MIM: 604145).

#### Diagnoses with implications for treatment

In 7 cases (Additional file 2: Table S3: ID 4, 6, 9, 10, 11, 23, 51) (13%), identifying the underlying genetic cause had immediate consequences for the treatment or management.

**Fig. 1:**
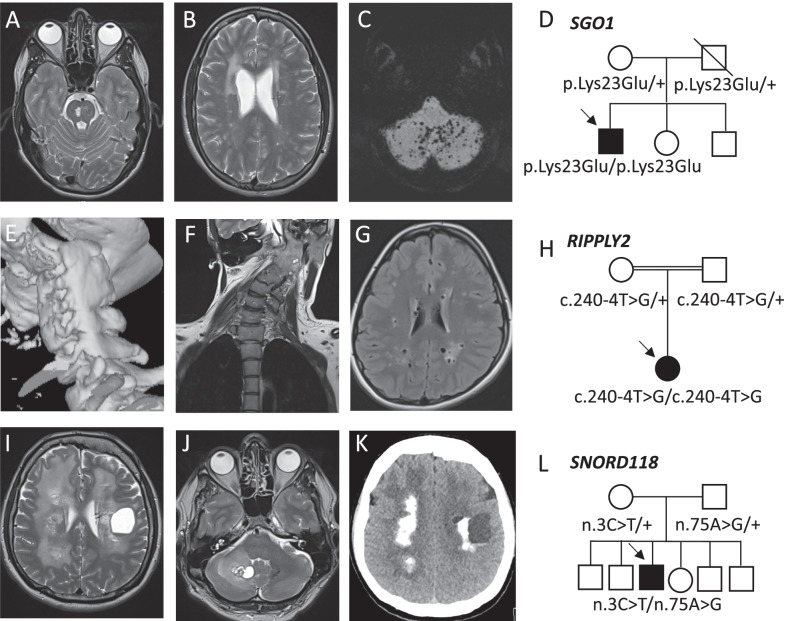
(Neuro)imaging of patients diagnosed with a rare disease which led to expansion of the phenotypic spectrum or which was associated with therapeutic changes. **A, B** T2- weighted MRI images of a patient diagnosed with CAID showing pontine and periventricular hyperintensities. **C** MRI image with maximum intensity projection (MIP) showing diffuse cerebellar microbleeds. **D** Pedigree of the CAID patient. He is the first child of non-consanguineous parents with a homozygous pathogenic *SGO1* variant (p.Lys23Glu). Both parents are heterozygotes. **E** 3D-reconstruction of CT-scan imaging of the cervical spine revealing a unilateral fusion of the cervical vertebrae C1-C6 in a patient diagnosed with spondylocostal dysostosis 6. **F** Coronal MRI image showing torticollis. **G** Axial MRI Flair image showing enlarged perivascular spaces and white matter lesions. **H** Pedigree of the patient with consanguineous parents and a homozygous *RIPPLY2* variant (c.240-4T>G). **I, J** T2-weighted MRI images of patient 4 revealing the presence of a large cyst in the left cerebral and the right cerebellar hemisphere and diffuse periventricular white matter hyperintensities. **K** CT-scan axial section showing diffuse cerebral calcifications. **L** Pedigree of the LCC patient. He is the only affected child of non-consanguineous parents and is compound heterozygous for two variants in *SNORD118* (n.3C > T; n.75A > G). Both parents were heterozygous for one pathogenic variant

Two independent male patients (patient 4 (Fig. 1I–L), 11) presenting with intellectual disability, epilepsy and movement disorders, were diagnosed with leukoencephalopathy with calcifications and cysts (MIM: 616663) due to compound heterozygous pathogenic variants (n.74G>A; n.*10G>A and n.3C>T; n.75A>G) in small nucleolar RNA *SNORD118* (box C/D snoRNA U8), required for ribosomal biogenesis and function. Treatment with anti-VEGF antibody bevacizumab was shown to have beneficial effects on extrapyramidal symptoms and cyst size on brain MRI [21]. We started VEGF inhibition in patient 4 which led to a significant cyst size reduction, though long-term clinical effects remain to be determined.

Patient 9 was referred to UD-PrOZA because of unexplained developmental delay, tremor, dyspraxia, and hypergonadotropic hypogonadism. After a diagnostic odyssey of 18 years including serial targeted genetic testing, she was diagnosed with galactosemia due to a homozygous pathogenic variant (p.Gln188Arg) in *GALT* (Galactose-1-Phosphate Uridydyltransferase) (MIM: 230400). Restriction of galactose intake and replacement of milk products by lactose-free formulas was advised with favorable effect on our patient’s symptoms and performance level.

Patient 6, a young female with a history of androgenetic alopecia presenting with pellagra-like dermatitis secondary to sun exposure, was diagnosed with Hartnup disease (MIM: 234500), caused by compound heterozygous variants in *SLC6A19* (p.Asp173Asn, p.Glu519Gly). Elevated excretion of neutral amino acids in urine confirmed the pathogenicity of these variants. A protein rich diet, avoidance of excessive exposure to sunlight and the intake of nicotinamide supplements was started.

Patient 10, a young male with a history of motor developmental delay and a diagnosis of autism spectrum disorder, was referred to UD-PrOZA because of subacute bilateral vision loss due to bilateral optic neuritis. Biochemical analysis showed severely decreased biotinidase enzyme activity (< 0.01 nmol/min/mL). Genetic testing of the *BTD* gene revealed compound heterozygosity for pathogenic variants (p.Gly36Ser, p.Cys425Arg), thereby confirming the diagnosis of biotinidase deficiency (MIM: 253260). After initiation of biotin supplements, significant improvement of vision and motor skills was seen. A more detailed description of this case has been published recently [22].

Patients 23 and 51 are discussed in other sections.

#### Expanding the phenotypic spectrum

Inherent to rare diseases is the incomplete ascertainment of their phenotypic spectrum due to the small size of published patient cohorts. In 4 cases (8%) (Additional file 2: Table S3: ID 8, 18, 21 and 48) we were able to expand the phenotypic spectrum of the disease.

Patient 8 is the third child of healthy non-consanguineous parents, with a medical history of recurrent intracerebral hemorrhages with diffuse microbleeds and periventricular and pontine hyperintensities on brain MRI, was referred to UD-PrOZA by his general practitioner (Fig. 1A–D, Additional file 2: Table S3). Additionally, the patient suffered from recurrent paralytic ileus and hyperpigmented skin lesions. WES revealed the presence of a homozygous missense variant in the *SGO1* gene (p.Lys23Glu), a known pathogenic founder mutation associated with autosomal recessive chronic atrial and intestinal dysrhythmia (CAID syndrome, MIM: 616201) [23]. *SGO1* encodes shugoshin-like 1 protein, which has a crucial function in the mitotic cell cycle by providing a link between the sister centromere cohesion complex and microtubules at kinetochores. Patients typically present with sick sinus syndrome requiring cardiac pacemakers and intestinal myogenic and neurogenic pseudo-obstruction. Recently, a novel association was reported between CAID syndrome and cerebral small vessel disease [24]. It was hypothesized that this cerebral vascular phenotype is a consequence of upregulated TGF-β signaling leading to vessel wall fibrosis and premature senescence of cerebellar arterioles due to mitotic dysregulation [23, 24]. Our patient is the third CAID patient in the literature in which cerebral small vessel disease is reported. This novel association might have important consequences, since a majority of the patients with cardiac arrythmias is treated with anticoagulation therapy, increasing the risk of cerebral hemorrhages.

A young female of Tunisian origin (patient 18), born from consanguineous parents (Fig. 1H), was referred because of disabling chronic migraine and abnormalities on brain MRI including widened Virchow-Robin spaces and periventricular white matter hyperintensities (Fig. 1G). Additionally, she has a torticollis due to a congenital unilateral fusion of cervical vertebrae C1-C6 (Fig. 1E, F). WES identified a homozygous splice region variant in *RIPPLY2* (c.240-4T>G) (Additional file 2: Table S3), a known founder mutation associated with spondylocostal dysostosis 6 (MIM: 616566) [25]. Although a clear effect on splicing in postnatal tissue has never been proven due to the embryonic expression of this gene, we classified this gene as likely pathogenic since in the literature, seven patients have been reported presenting with congenital posterior cervical spine malformations in which this splice region mutation was identified in homozygous or compound heterozygous state [24]. In only one patient a Klippel-Feil syndrome (fusion of the cervical vertebrae) was reported, all other patients presented with agenesis of the posterior vertebral elements. Whether this variant could also be explanatory for the findings on brain MRI, remains elusive and the possibility of a second yet unidentified molecular cause has to be taken into account.

Patient 48 was referred for genetic testing because of a dilated cardiomyopathy. Additionally, he suffers from chronic back pain for which he is treated with a neurostimulator after failed back surgery. He complained of proximal muscle weakness and myalgia, predominantly of the upper limbs. WES revealed compound heterozygous pathogenic variants in *SELENON* (p.Val447Met, c.997_1000del), associated with rigid spine muscular dystrophy 1 (MIM: 602771). Selenoprotein N, an endoplasmic reticulum glycoprotein, plays a key role in redox-based calcium homeostasis and protects the cell against oxidative stress. In the literature, one patient with pathogenic bi-allelic *SELENON* variants and dilated cardiomyopathy was reported [26]. Other reported *SELENON*-related cardiac abnormalities include cor pulmonale and valvular disease. As such, we establish dilated cardiomyopathy as a feature being part of the rigid spine muscular dystrophy 1 phenotypic spectrum.

Patient 21 is discussed elsewhere.

#### Use of model organisms

Small model organisms (*Drosophila melanogaster/ Danio rerio*) provide us with unique in vivo readouts helping us to understand the biological relevance of variants of unknown significance identified through NGS techniques.

**Fig. 2:**
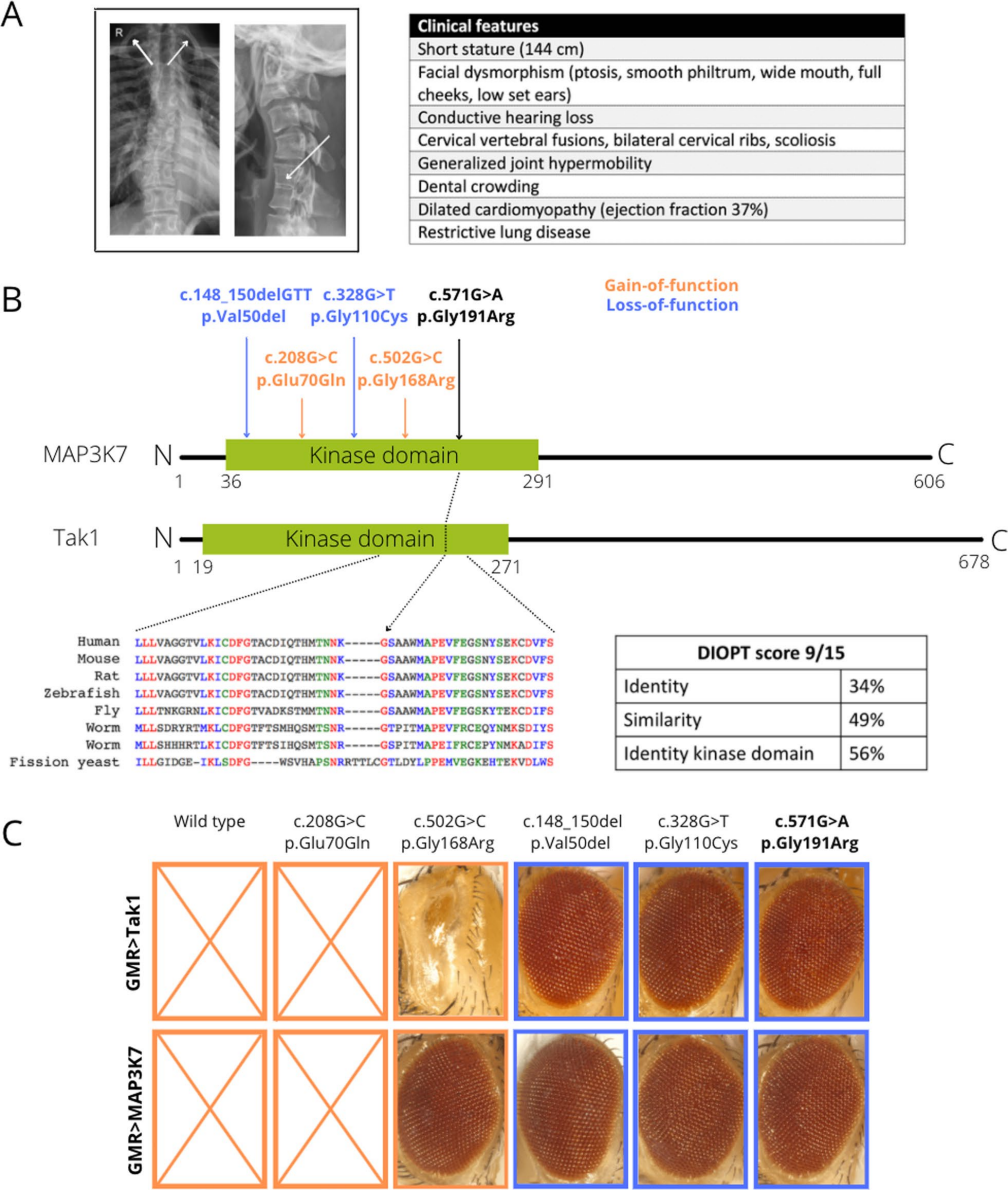
Variant modeling in *Drosophila* establishes *MAP3K7* p.(Gly191Arg) as a novel pathogenic LoF variant causing CSCFS. **A** Posteroanterior view of a chest X-ray showing scoliosis and bilateral cervical ribs. Lateral view of a cervical spine X-ray shows a fusion of C5 and C6. An overview of all clinical features is provided. **B** The MAP3K7 and Tak1 protein contain an evolutionary conserved kinase domain. Two of the known GoF variants (associated with FMD2) are depicted in orange, two of the known LoF variants (associated with CSCFS) are represented in blue. Our new variant (c.571G>A, p.Gly191Arg), depicted in black, is located in de kinase domain and replaces a highly conserved glycine for an arginine residue. **C** Overexpression of wild-type *Drosophila* Tak1 and human MAP3K7 in the *Drosophila* eye using a GMR driver resulted in early pupal lethality. Overexpression of fly Tak1 and human MAP3K7 with GoF variant p.Glu70Gln fully recapitulated the phenotype of wild-type overexpression. With the p.Gly168arg variant a discrepancy between the human and fly protein was noted. Overexpression of the fly Tak1 variant resulted in a severe eye phenotype whereas overexpression of the human variant did not visibly affect the eye, pointing towards a milder GoF effect compared to the other GoF mutation. Overexpression of known LoF variants p.Val50del and p.Gly110Cys in human and fly proteins lost the wild-type eye phenotype. Overexpression of our novel variant p.Gly191Arg resulted in a normal eye thereby establishing the LoF nature of this variant

Patient 21 was referred because of small stature, dysmorphic facial features, fused cervical vertebrae, dilated cardiomyopathy, conductive hearing loss and joint hyperlaxity (Fig. 2A). WES identified a previously unreported de novo heterozygous missense variant in the *MAP3K7* gene (p.Gly191Arg), located in the conserved catalytic kinase domain of the enzyme (Fig. 2B, Additional file 2: Table S3). *MAP3K7* encodes the transforming growth factor-β-activated kinase 1 (TAK1) which plays an important role in the cascade of cellular responses evoked by changes in the environment. Pathogenic variants in this gene cause autosomal dominant cardiospondylocarpofacial syndrome (CSCFS, MIM: 157800) and frontometaphyseal dysplasia 2 (FMD2, MIM: 61737), depending on the loss- (LoF) or gain-of-function (GoF) nature of the causal variant. The p.Gly191Arg variant is not present in gnomAD, has deleterious in silico predictions and replaces a highly conserved glycine residue for an arginine residue, an amino acid with different physicochemical properties (Fig. 2B). In order to investigate the LoF or GoF mechanism of this novel variant we used *Drosophila* to assess kinase activity. It has been shown that hyperfunction of the TAK1 kinase in the fly eye, results in a rough eye phenotype [27]. We replicated these findings and observed that eye-specific overexpression of wild-type fly and human TAK1 resulted in a severe eye phenotype, even causing pupal lethality (Fig. 2C). Next, we exploited these findings to assess the effect of known variants on the kinase activity [28, 29]. Overexpression of known pathogenic LoF variants (c.148_150delGTT, p.Gly110Cys), completely reversed the phenotype, whereas overexpression of known GoF variants (p.Glu70Gln, p.Gly168Arg) preserving kinase activity mimic the wild-type phenotype. It has been shown that patients heterozygous for the p.Gly168Arg variant present with a less severe FMD phenotype, which is reflected in a less severe eye phenotype or even no phenotype when overexpressing the fly and human TAK1 with this variant [30]. Overexpression of fly and human TAK1 with our novel variant (p.Gly191Arg) resulted in seemingly healthy flies with normal eyes and normal life spans, as such establishing a LoF nature of this missense variant. This case illustrates the use of small model organisms for quick variant modeling in diagnostics of rare disease. Furthermore, this case expands the phenotypic spectrum of the CSCF syndrome, since cardiomyopathy is a feature which has not been associated with this syndrome before.

#### Identification of novel disease genes

UD-PrOZA contributed to the discovery of three new disease genes (Table S3: ID 3, 23, 51) by performing hypothesis-free NGS testing and using the GeneMatcher [6] platform to get in touch with other researchers that identified variants in the same candidate genes.

A male patient (patient 3) with severe intellectual disability, epilepsy, absence of speech, ataxia, choreoathetosis and generalized dystonia was referred by the neurologist for genetic testing. The first signs of motor regression started at the age of 5 years and worsened over time, resulting in wheelchair dependence since the age of 28. Trio-WES analysis detected a de novo heterozygous nonsense variant (p.Gln126Ter) in the *IRF2BPL* gene (Interferon regulatory factor 2 binding protein-like), which encodes a member of the IRF2BP family of transcriptional regulators (Table S3). The gene had not been associated with a phenotype in humans. The present case is part of the initial study describing LoF variants in *IRF2BPL* as the cause of the novel disorder ‘Neurodevelopmental disorder with regression, abnormal movements, loss of speech and seizures’ (MIM: 618088) [31].

Patient 23, a female patient born from consanguineous Turkish parents, was referred to UD-PrOZA for genetic testing for an unexplained partial lipodystrophy phenotype. She and two female siblings presented with lipoatrophy of the limbs and trunk, dyslipidemia, insulin resistant diabetes and hepatosteatosis. Through WES and WGS, we identified a homozygous 5092 bp deletion encompassing exon 2 of the *PLAAT3* gene within a shared homozygous region. This gene encodes an adipocyte specific phospholipid modifying enzyme which we showed to serve as an important regulator of PPAR-γ activity, the master regulator of adipocyte differentiation and function. The detailed study in which we establish *PLAAT3* deficiency as a novel cause for autosomal recessive familial partial lipodystrophy is described elsewhere (https://doi.org/10.1101/2021.04.15.439941v1.article-metrics).

Patient 51, a female patient born from consanguineous parents, was referred because of unexplained subacute anhedonia, hallucinations, motor problems, dysarthria, insomnia and diffuse leukoencephalopathy on brain MRI. WES identified a homozygous nonsense variant (p.Arg183Ter) in the *ACMSD* gene, encoding the enzyme aminocarboxymuconate-semialdehyde-decarboxylase, involved in the catalytic breakdown of tryptophan into NAD^+^. The enzyme is located at a key branch-point of the pathway, limiting the production of the neurotoxin quinolinic acid, which has excitotoxic and pro-inflammatory properties. SNPs and heterozygous rare variants in *ACMSD* have been associated with neurodegenerative diseases such as Parkinson’s disease [32, 33]. Moreover, this enzyme was appointed as a potential therapeutic target for neurodegenerative diseases. The homozygous nonsense variant identified in this patient is predicted to induce nonsense mediated decay and in silico predictions suggest pathogenicity. In population database gnomAD no homozygous LoF alleles have been reported. Additionally, using GeneMatcher [6] we identified a second pediatric patient with a severe neurological phenotype and a homozygous LoF variant in *ACMSD*. The variants identified in the two patients are predicted to induce nonsense mediated decay and as such completely abrogate the aminocarboxymuconate-semialdehyde-decarboxylase enzyme activity. Further investigations and functional analyses are currently ongoing to elaborate on this novel disease gene. We hypothesize that a diet poor in tryptophan might have beneficial effects on the neurological features seen in both patients.

#### Unsolved cases

In the majority of the cases in which an underlying rare hereditary disorder was suspected, the molecular cause remained elusive, despite thorough genetic testing. Below we describe as an example six patients from four families in which a monogenic cause is highly likely, but we were unable to pinpoint the disease-causing variant.

Three presumably unrelated pediatric patients from Bulgarian origin (patient A, B and C) were referred to UD-PrOZA because of an unexplained syndromic entity characterized by congenital neurosensorial deafness, agenesis of the basal ganglia, severe developmental delay and laryngomalacia (Figure S3A). Two out of the three patients had an affected deceased sibling suggesting autosomal recessive inheritance. Molecular karyotyping and WES could not reveal the molecular cause.

Three siblings born from consanguineous parents presented with a similar phenotype characterized by hypotonia, global developmental delay, facial dysmorphism (large ears, exaggerated Cupid’s Bow), peripheral neuropathy and conjunctival telangiectasia (Figure S3B). Although an underlying autosomal recessive disorder is highly likely considering the consanguinity, we were not able to identify the molecular cause after homozygosity mapping, WES and molecular karyotyping.

## Discussion

In this study we present UD-PrOZA, a novel multidisciplinary diagnostic platform, established at the Ghent University Hospital in Belgium in May 2015. UD-PrOZA aims to facilitate the diagnostic process for adult patients with suspected rare diseases by integrating a multidisciplinary clinical evaluation, state-of-the-art genomic technology and data sharing. Compared to other initiatives, UD-PrOZA is unique in terms of both modus operandi as well as the main field of interest. Unlike other initiatives, UD-PrOZA is a monocentric platform and accepts only referrals from other physicians, which has proven an efficient first filter before approval of the application. Comparable rare diseases initiatives predominantly focus on pediatric unsolved cases, whereas UD-PrOZA is mainly involved in diagnosing adult-onset rare diseases and adult patients with longstanding unexplained disease, that often already started at a young age. An overview of other rare disease programs and initiatives is provided in Table 3. With diagnostic rates between 35 and 67% [14–19], other initiatives outscore UD-PrOZA which was able to diagnose 18% of all accepted cases. This discordance can be most likely attributed to the age difference of the patient cohorts since other initiatives focus on pediatric cases often presenting with severe neurodevelopmental phenotypes and/or congenital malformations [14, 18]. The a priori chance of identifying an underlying molecular cause is higher in children, presenting with more severe and typical phenotypes, as opposed to adults which often present with an atypical or attenuated phenotype [9, 34]. Nevertheless, with a diagnostic rate of 18% we show that advanced age should not be a counter-argument to initiate exome-based genetic testing. We showed that the presence of objective disease signs is an important criterium to evaluate the likelihood of an underlying rare disease and a prerequisite to initiate genetic testing. Currently, exome-based gene panel testing is done in our center in a diagnostic setting in adults. Analysis of the entire Mendeliome however, is limited to prenatal, neonatal and research settings.

**Table 3:** Comparison to other rare disease initiatives

Name	Referred patients	Monocentric/multicentric	Accepted patients*	% of patients < 18y	Diagnostic rate	Phenotypes	Reference
Initiative on Rare and Undiagnosed Disease in Japan (IRUD)	5359	Multicentric	4205	*NA*	42.9%	Diverse	Takahashi et al. [15]
Undiagnosed Disease Network (UDN)	1519	Multicentric	601	57%	35%	Diverse	Splinter et al. [14]
Program for undiagnosed rare diseases (UD-PrOZA)	692	Monocentric	329	6.7%	18%	Diverse	*This study*
Singapore Undiagnosed Disease Program	NA	Multicentric	196	90%	37.2%	Global developmental delay/ Congenital malformations	Bhatia et al. [16]
The Korean undiagnosed diseases program (KUDP)	NA	Multicentric	72	94.8%	38.9%	Diverse	Kim et al. [17]
SpainUDP	NA	Multicentric	30	74.1%	67%	Diverse	López-Martín et al. [18]
The Italian Undiagnosed Rare Diseases Network (IURDN)	110	Multicentric	13	31% (92% onset < 18y)	53.8%	Diverse	Salvatore et al. [19]
Undiagnosed Diseases Program – Western Australia (UDP-WA)	NA	Multicentric	NA	NA	NA	NA	Baynam et al. [46]
National Network to Collaborate on Diagnosis and Treatment of Rare Diseases China	NA	Multicentric	NA	NA	NA	NA	Ren et al. [47]

*NA*: not available

^*^In which genotyping and phenotyping were finished

The mean time interval between symptom-onset and diagnosis was 19 years. This diagnostic delay is associated with numerous doctor visits and investigations, putting an enormous burden on health care budgets. More importantly, these patients remain in limbo concerning prognosis and the recurrence risk for family members, inducing high levels of anxiety. This striking diagnostic delay emphasizes the need for alternative approaches to diagnose rare diseases.

Inherent to the early implementation of WES-based diagnostic testing is the identification of incidental findings. In 7% of the patients in whom WES was performed, actionable incidental findings were reported, which is in accordance with the frequency reported in the literature ranging from 2.5 to 12% [35–37].

This study and the proposed diagnostic platform have several limitations. The restriction of referral by healthcare providers only, can be a limiting factor for patients to be evaluated by UD-PrOZA. However, it has been shown in previous studies that a large proportion of patients who directly contact centers for rare undiagnosed diseases, presented with subjective complaints (pain symptoms, fatigue, irritable bowel syndrome) and were never evaluated at the tertiary level [38]. Furthermore, we did not evaluate the cost-effectiveness of our diagnostic approach in which exome-based genetic testing is initiated in an early phase of the diagnostic process. Although the implementation of NGS genetic testing for suspected monogenic disorders has shown to be cost-effective in children, comparable data in adults is lacking [9, 10, 39, 40].

The diagnostic yield of 22% after genetic testing is probably an underestimation, taken into account the frequent identification of class 3 variants, potentially associated with the patient phenotype. Further functional analyses and/or data sharing efforts are currently undertaken to address the potential causality of these variants. A diagnostic yield of 22% implies that in the majority of the cases in which a monogenic disease is suspected, we could not identify the underlying molecular cause. There are several potential reasons why we were not able to identify the causal mutation. First, there might not be a monogenic cause underlying the phenotype of the patient. In some cases, Mendelian disorders can be difficult to distinguish from multifactorial diseases (e.g., stroke, Parkinson’s disease, auto-inflammatory syndromes, etc.). Second, pathogenic variants in genes which have not yet been established as disease genes (OMIM) might be underlying the phenotype emphasizing the importance of periodic reanalysis of exome data [41]. Third, the technique of short read whole exome sequencing has its shortcomings and is not suited to detect repeat expansions or variation in non-coding sequences. Additionally, coding variants might be missed due to insufficient coverage or low-grade mosaicism.

As a part of its future perspectives, UD-PrOZA is now implementing WGS and RNA sequencing to identify pathogenic variants in non-coding sequences. In a UDN patient cohort, 24% of all genetic diagnoses were based on WGS technology [14]. We predict that performing WGS in unexplained cases will significantly increase the diagnostic rate since it was shown in the literature that WGS has a higher diagnostic yield compared to WES across a variety of clinical indications [42]. In studies comparing WGS with conventional genetic testing (i.e. gene panel testing) and exome sequencing, a diagnostic increase was seen between 14.5 and 17% in favor of WGS [43, 44]. The missed diagnoses included structural and non-exonic sequence variants not detected with WES, but also variants previously missed due to technical problems such as incomplete coverage. Interestingly, it was shown that 30% of the positive cases identified by WGS in a WES-negative cohort, could also have been identified by reanalyzing the WES data resulting in an overestimation of the clinical utility of WGS [45]. That is why UD-PrOZA will systematically reanalyze the existing WES and WGS data as knowledge on rare diseases quickly expands, including the continuous discovery of new diseases, genes and pathogenic variants [41]. Lastly, UD-PrOZA is making an effort to facilitate the online referral platform and to integrate artificial intelligence to improve case selection and diagnostic yield.

## Conclusion

UD-PrOZA offers an innovative platform to diagnose rare diseases in adults. With a total diagnostic rate of 18%, this multidisciplinary approach, combined with early implementation of exome-based genetic testing and close collaboration with research facilities has proven to be an efficient strategy to diagnose previously unrecognized rare diseases.

## Supplementary Information

**Additional file 1.** Supplementary methods, figures and tables (Figures S1, S2, S3 and Tables S1, S2).

**Additional file 2**. An overview of all genetic diagnoses is provided (Table S3).

## Data Availability

The data that support the findings of this study are available on request from the corresponding author.
