# Supplementary material for: Shortcutting the diagnostic odyssey: the multidisciplinary Program for Undiagnosed Rare Diseases in adults (UD-PrOZA)
Source: Orphanet J Rare Dis. 2022 May 23;17:210. doi: 10.1186/s13023-022-02365-y (PMC9128245; doi:10.1186/s13023-022-02365-y)
Supplement: Supplementary file 1 — Additional file 1. Supplementary methods, figures and tables (Figures S1, S2, S3 and Tables S1, S2). [file 13023_2022_2365_MOESM1_ESM.docx]

**Shortcutting the diagnostic odyssey: the multidisciplinary Program for Undiagnosed Rare Diseases (UD-PrOZA)**

Nika Schuermans,^1,2*^ Dimitri Hemelsoet,^3*^ Wim Terryn,^4^ Sanne Steyaert,^5^ Rudy Van Coster,^6^ Paul Coucke,^1,2^ Wouter Steyaert,^7^ Bert Callewaert,^1,2^ Elke Bogaert,^1,2^ Patrick Verloo,^6^ Arnaud Vanlander,^6^ Elke Debackere,^1,2^ Jody Gijsels,^1,2^ Pontus LeBlanc,^1,2^ Hannah Verdin,^1,2^ Leslie Naesens,^8,9^ Filomeen Haerynck,^8^ Steven Callens,^5^ Bart Dermaut,^1,2,#^ Bruce Poppe,^1,2,#^ for UD-PrOZA

**Supplementary data**

1. **Supplementary methods**
2. **Supplementary figures**
3. **Supplementary tables**

**Supplementary methods**

*UD-PrOZA workflow*

The UD-PrOZA workflow is summarized in figure S1. Patients can be referred to UD-PrOZA by health care providers only. All referred patients with an unexplained medical condition despite exhaustive medical evaluation are considered by the UD-PrOZA team. There are no predefined criteria to decide upon eligibility for further UD-PrOZA evaluation. The medical records are reviewed by a multidisciplinary team of clinicians who evaluate the likelihood of an underlying rare disease. Eligible patients are invited to a multidisciplinary consultation where a detailed personal and familial medical history is taken and a physical examination is done, after which a tentative diagnosis is made, or additional investigations are requested. In the majority of the cases genetic testing is initiated, which can be targeted gene testing in case the phenotype is suggestive for a specific Mendelian disease, or can be WES-based, in case of genetic heterogeneity. To elaborate on variants of unknown significance or on potential new disease genes, PrOZA makes use of data sharing portals GeneMatcher^(1)^ and PhenomeCentral^(2)^. For some cases collaboration with model animal facilities or the proteomics/metabolomics/lipidomics core is engaged to determine the involvement of certain genes or variants in a specific phenotype. Some unsolved cases were submitted for further evaluation by Solve-RD.

*Genetic testing*

Targeted genetic testing: Targeted mutation or gene analysis is performed through Sanger sequencing making use of the Big DYE Terminator Cycle Sequencing Ready Reaction Kit (Applied Biosystems) after PCR amplification of the sequence of interest. For more comprehensive gene screening, Multiplicom’s MASTR (Multiplex Amplification for Specific Targets for Resequencing) assays are used to enable multiplex PCR amplification, followed by amplicons sequencing on the Illumina Miseq.

Homozygosity mapping: Genotyping for homozygosity mapping was performed using the 200K genome-wide HumanCytoSNP-12 v2 BeadChip single nucleotide polymorphism (SNP) array (Illumina, San Diego, CA). The position of the probes was based on NCBI build GRCh37. Shared homozygous regions were detected using the PLINK algorithm (v1.07, default settings).

Whole exome sequencing (WES): WES was done on the Illumina Hiseq 3000 and the Novaseq 6000 Platform after enrichment of gDNA with SureSelectXT Low Input Human All Exon v6 and v7 (Agilent Technologies). The BWA-MEM 0.7.17 algorithm was used for read mapping against the human genome reference sequence (NCBI, GRCh37.p5/hg19), duplicate read removal, and variant calling. Variant calling and filtering were done using Seqplorer, an in-house developed tool for the analysis of WES data. The position of the called variants is based on NCBI build GRCh38. A minimum of 90% of the interrogated genes have a coverage of >20x. Variant classification was done according to the ACMG guidelines. Potential CNVs were called using ExomeDepth, an algorithm which uses WES data to detect read depth differences in coding regions.

Molecular karyotyping was done by means of low-pass whole-genome sequencing (CNV-seq) on the Illumina Novaseq 6000 with a genome wide resolution of 100kb (GRCh38).

Sequencing of mitochondrial DNA: mtDNA is amplified through Long Range PCR, after which the amplicon is fragmented into approximately 220 base pair fragments. The library preparation is performed according to the instructions of the producer (KAPA Biosystems). The fragments are sequenced on the Illumina Novaseq 6000 platform. The nomenclature is based on the rCRS NC_012920.1 sequence. The detection limit for heteroplasmy of >3% is guaranteed.

*Drosophila MAP3K7* variant modeling

*Fly stocks and maintenance*

Drosophila melanogaster strains were maintained on standard Nutrifly formula food, yellow cornmeal, agar (type II), corn syrup solids, inactive nutritional yeast, soy flour (Genesee Scientific) in a 12 hr light/dark rhythm temperature controlled. The w1118 (Canton-S10) line was used as control and the GMR-GAL4 line (Bloomington stock center) was used to drive expression in the fly eye. Crosses for adult offspring frequencies and phenotypic data were performed at 25°C. To generate UAS-Map3K7 and UAS-TAK1 fly lines, the coding region of those genes was subcloned in the pUAST-attB backbone (GenScript Biotech, Netherlands) allowed the generation of transgenic fly lines by targeted insertion into the 68A4 attP locus on the third chromosome (GenetiVision, USA).

*Missexpression studies: offspring quantification and external eye phenotype*

For each cross the collected offspring was divided by sex and the genotypes were counted according to the balancers. The offspring ratio was determined by: counted offspring/expected offspring. Adult flies were anesthetized with CO2 and images were taken with a zoom stereo microscope (Leica Z16APO).

**References**

1. Sobreira N, Schiettecatte F, Valle D, Hamosh A. GeneMatcher: a matching tool for connecting investigators with an interest in the same gene. Hum Mutat. 2015;36(10):928-30.

2. Buske OJ, Girdea M, Dumitriu S, Gallinger B, Hartley T, Trang H, Misyura A, Friedman T, Beaulieu C, Bone WP, Links AE, Washington NL, Haendel MA, Robinson PN, Boerkoel CF, Adams D, Gahl WA, Boycott KM, Brudno M. PhenomeCentral: a portal for phenotypic and genotypic matchmaking of patients with rare genetic diseases. Hum Mutat. 2015;36(10):931-40.

**Supplementary figures**

**Figure S1.** Overview of the UD-PrOZA workflow

**Legend:**

Patients with the suspicion of an underlying rare disease are referred by a healthcare provider after which their medical record is reviewed by the multidisciplinary team (n=692). If a rare disease is likely, the patients are invited for a multidisciplinary consultation (n=329) for gathering additional clinical information, physical examination and the initiation of paraclinical investigations. In some patients, a clinical diagnosis is made (n=7), in others genetic testing is initiated (n=237). In 22% (53 out of 237), genetic testing led to a molecular diagnosis, including novel genotype-phenotype correlations (n=4) and the identification of novel disease genes (n=3). To elaborate on these findings and on variants of unknown clinical significance, functional testing and data sharing is required (n=13). In 78% (184 out of 237), no underlying cause could be identified. These are all candidates for future WGS/transcriptomics analysis to identify potentially causal mutations in non-coding sequences.

**
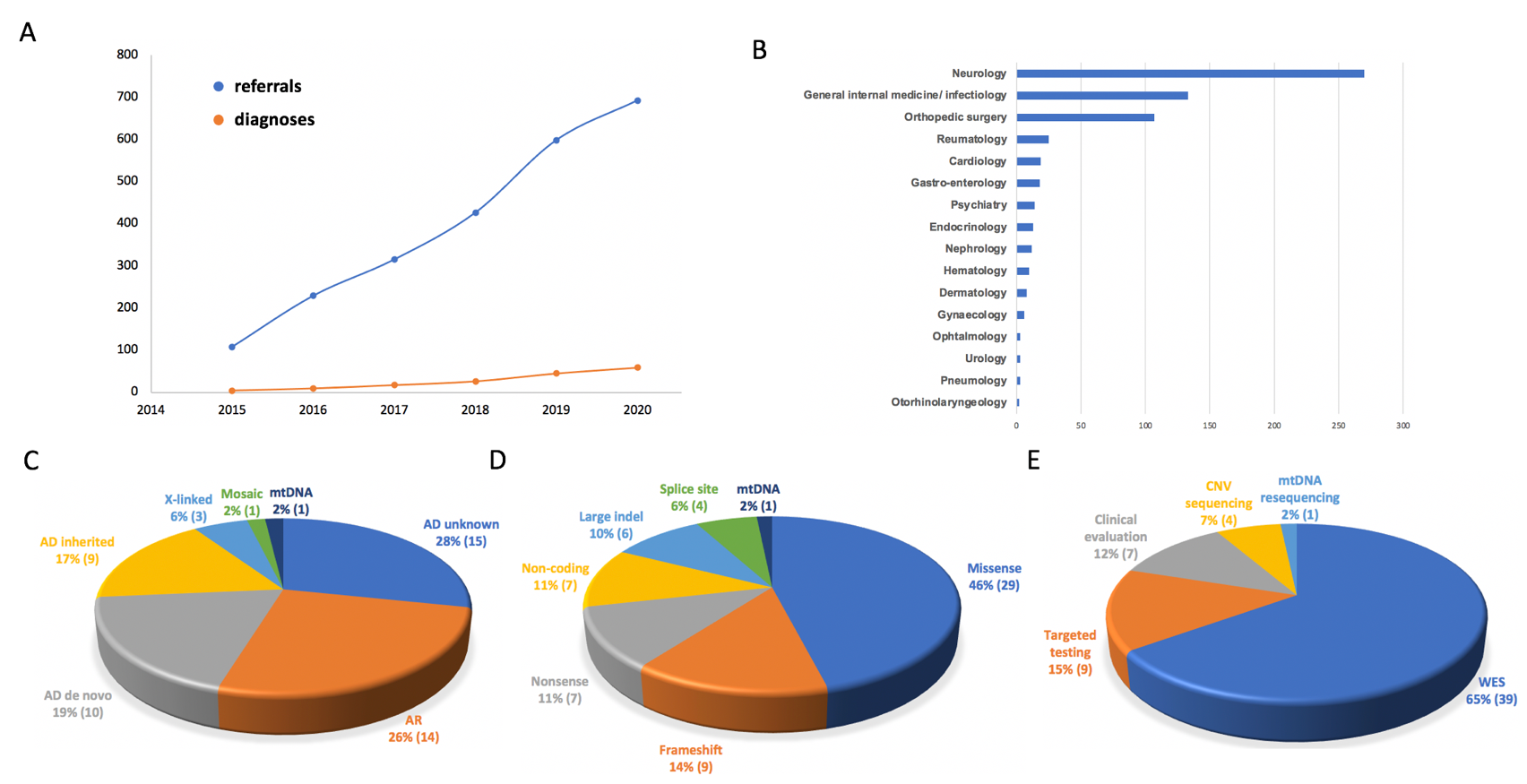
Figure S2.** Graph of referrals and diagnosed cases over time, referrals per medical discipline, pie charts of inheritance mode, mutation type and diagnostic tool.

**Legend:**

**A** Cumulative referrals (blue) and diagnoses (orange) per year. **B** Referrals (absolute) per medical discipline. **C-E** Pie chart distributions of inheritance mode, variant type and diagnostic tool used to identify the diagnosis.


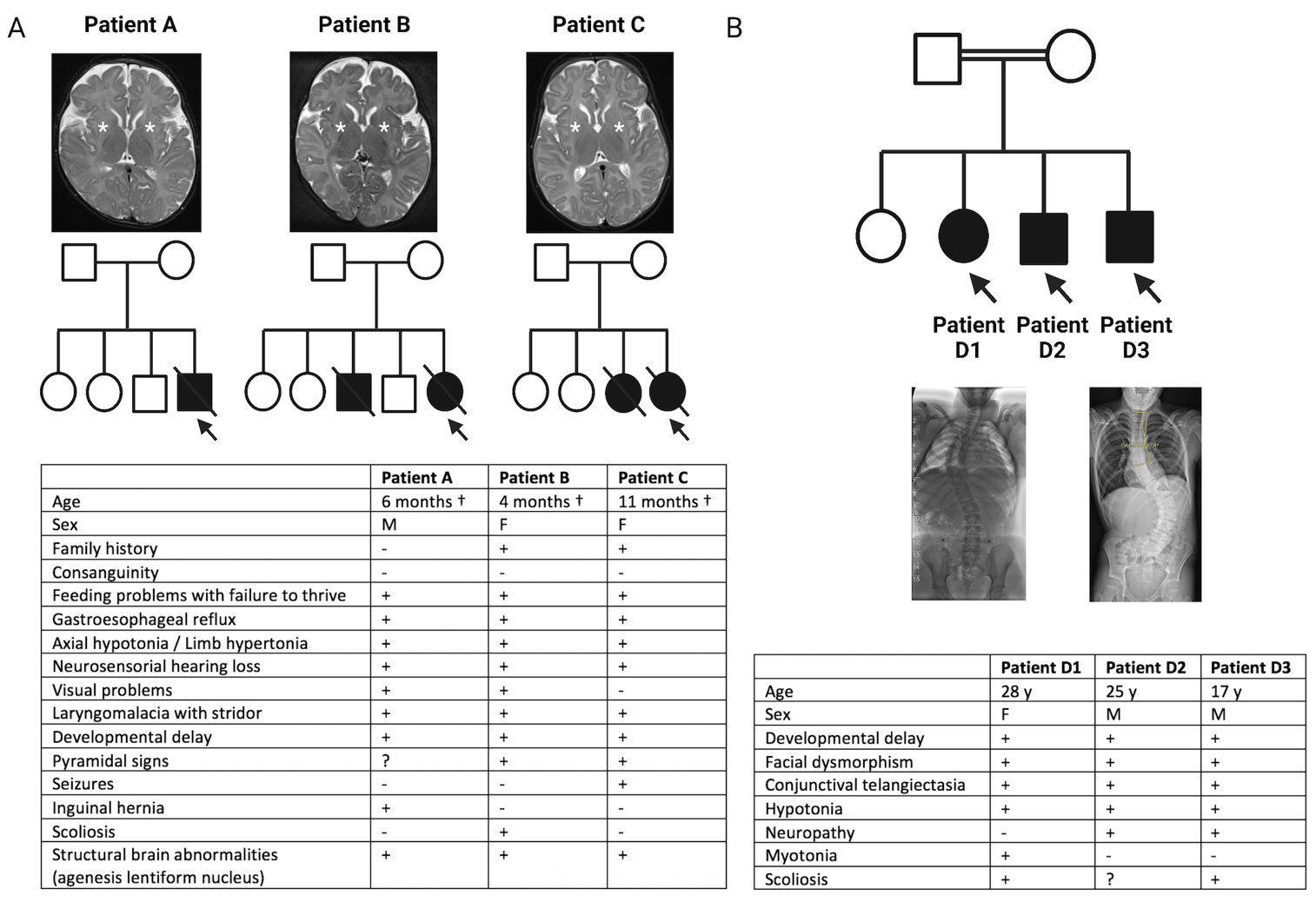
**Figure S3.** Unsolved cases

**Legend:**

**A** Axial T2-weighted brain MRI images of unrelated pediatric patients A, B and C. The bilateral absence of the lentiform nuclei is indicated by an asterisk (*). Pedigrees of patients A, B and C; probands are indicated by an arrow. Overview of phenotypic features of patients A, B and C. **B** Pedigree of a consanguineous family with three affected siblings (patients D1, D2 and D3), indicated by arrows, and one healthy sibling. Posteroanterior view of full spine X-rays of patients D1 and D3 showing severe scoliosis. Overview of phenotypic features of patients D1, D2 and D3.

**Supplemental tables**

**Table S1.** Overview of all clinical diagnoses

| ID | Sex | Phenotype/Medical history | Additional testing | Diagnosis |
| --- | --- | --- | --- | --- |
| 54 | M | Aortic valve stenosis, third-degree atrioventricular block, neurosensorial deafness, axonal polyneuropathy, episodic vertigo, multinodular goiter | Ophthalmological examination: salt and pepper pigmentary retinopathy, lens opacities | **Congenital rubella syndrome** |
| 55 | M | Syrian origin, developmental delay, severe intellectual disability, small stature, facial dysmorphism | Blood analysis: TSH, FT3, FT4, conventional karyotyping: trisomy 21 | **Congenital hypothyroidism secondary to trisomy 21** |
| 56 | F | Fever, skin rash, polyarthritis, myalgia, pharyngitis, pericarditis, pleuritis, splenomegaly, elevated ESR, CRP and serum ferritin, granulocytosis | Blood analysis: neutrophils, ferritin, CRP | **Adult Still’s disease** |
| 57 | M | Dyspnea, cough, wheezing, allergic rhinitis, eczema, erythroderma, axonal peripheral polyneuropathy, eosinophilia (58% lab test 2016) | 46XY; FIP1L1-PDGFRA fusion absent | **Primary hypereosinophilic syndrome** |
| 58 | M | Arthritis, myalgia, urticaria, anemia, abdominal pain, nausea, vomiting, scleritis | Blood analysis: C1q, C1q auto antibodies | **McDuffie syndrome (hypocomplementemic urticarial vasculitis)** |
| 59 | M | Progressive muscle weakness and atrophy right hand (predominantly affecting C8-T1 musculature), absence of sensory deficits | MRI cervical spine, mtDNA sequencing/WES negative | **Hirayama disease (monomelic amyotrophy)** |
| 60 | F | Diffuse musculoskeletal pain, episodic fever, urticarial skin rash, and malabsorption after bariatric surgery | Hereditary fever gene panel analysis negative, favorable response to corticoid and antibiotic treatment | **BADAS (Bowel Associated Dermatosis Arthritis Syndrome)/ Blind loop syndrome** |

**Table S2.** Secondary findings

| **Secondary finding** | **OMIM phenotype (MIM number)** |
| --- | --- |
| ***PALB2*** c.2834+1G>T | Breast cancer, susceptibility to, AD (114480) |
| ***LDLR*** p.Gly343Cys | Hypercholesterolemia, familial, 1, AD/AR (143890) |
| ***BRCA1*** p.Glu733ThrfsTer5 | Breast cancer, susceptibility to, AD (114480) |
| ***MUTYH*** p.Tyr152Cys | Adenomas, multiple colorectal, AR (608456) |
| ***BRCA2*** p.His2090GlnfsTer9 | Breast cancer, susceptibility to, AD (114480) |
| ***CHEK2*** c.444+1G>A | Breast cancer, susceptibility to, AD (114480) |
| ***CHEK2*** c.1100del | Breast cancer, susceptibility to, AD (114480) |
| ***MYBPC3*** p.Gly235SerfsTer74 | Left ventricular noncompaction 10, AD (615396)  Cardiomyopathy, hypertrophic, 4, AD/AR (115197) |
| ***HOXB13*** p.Gly84Glu | Prostate cancer, hereditary, 9 (610997) |
| ***ATM*** p.Glu522IlefsTer43 | Breast cancer, susceptibility to, AD (114480)  Ataxia-telangiectasia, AR (208900) |
| ***HOXB13*** p.Gly84Glu | Prostate cancer, hereditary, 9 (610997) |
| ***ATM*** p.Asp841IlefsTer6 | Breast cancer, susceptibility to, AD (114480)  Ataxia-telangiectasia, AR (208900) |

**Table S3.** Overview of all genetic diagnoses

(See Additional file 1)
