# Supplementary material for: Shortcutting the diagnostic odyssey: the multidisciplinary Program for Undiagnosed Rare Diseases in adults (UD-PrOZA)
Source: Orphanet J Rare Dis. 2022 May 23;17:210. doi: 10.1186/s13023-022-02365-y (PMC9128245; doi:10.1186/s13023-022-02365-y)
Supplement: Supplementary file 2 — Additional file 2. An overview of all genetic diagnoses is provided (Table S3). [file 13023_2022_2365_MOESM2_ESM.docx]

| **ID** | **Sex** | **Patient phenotype** | **Onset** | **Delay (y)** | **Family History** | **Tool** | **Gene(s)** | **RefSeq** | **Variant(s)** | **ACMG criteria** | **Segregation** | **OMIM phenotype** | **Prevalence (Orphanet)** |
| --- | --- | --- | --- | --- | --- | --- | --- | --- | --- | --- | --- | --- | --- |
| 1 | F | Muscle weakness, myalgia, elevated CK | Adolescence | 3 | No | WES singleton | ***ANO5*** | NM_213599.2 | c.1213C>T,p.Gln405Ter;  c.191dup, p.Asn64LysfsTer15 | PVS1, PP5, PP3, PM2  PVS1, PP5, PP3 | Compound heterozygous | Muscular dystrophy, limb-girdle, autosomal recessive 12 (611307) | Unknown |
| 2 | F | ID, spastic paraparesis, central iron accumulation | Neonatal | 28 | No | WES singleton | ***WDR45*** | NM_007075.3 | c.902_906del, p.Phe301CysfsTer4^(a)^ | PVS1, PP3, PM2 | Heterozygous, de novo | Neurodegeneration with brain iron accumulation 5, X-linked dominant (300894) | <1/1.000.000 |
| 3 | M | ID, generalized dystonia, epilepsy, ataxia, chorea | Childhood | 36 | No | WES singleton | ***IRF2BPL*** | NM_024496.3 | c.376C>T, p.Gln126Ter^(b)^ | PVS1, PM2, PP3 | Heterozygous, de novo | Neurodevelopmental disorder with regression, abnormal movements, loss of speech, and seizures; autosomal dominant (618088) | Unknown |
| 4 | M | Hypokinetic dysarthria, brain cysts and calcifications | Adulthood | 6 | No | Targeted testing | ***SNORD118*** | NR_033294.1 | n.3C>T;  n.75A>G | PM2, PM3, PP4, PP5  PM2, PM3, PP4, PP5 | Compound heterozygous | Leukoencephalopathy, brain calcifications, and cysts; autosomal recessive (614561) | <1/1.000.000 |
| 5 | M | SCID | Neonatal | 26 | Yes | WES singleton | ***IL2RG*** | NM_000206.2 | c.598C>T, p.Gln200Ter^(a)^ | PVS1, PM2, PP3 | Hemizygous, maternally inherited | Severe combined immunodeficiency, X-linked; SCIDX1 (300400) | 1-9/100.000 |
| 6 | F | Androgenetic alopecia, pellagra-like dermatitis | Adolescence | 11 | No | WES singleton | ***SLC6A19*** | NM_001003841.2 | c.517G>A, p.Asp173Asn;  c.1556A>G, p.Glu519Gly^(a)^ | PS3, PM2, PP2, BP4, PM3  PS3, PM2, PP2, PP3, PM3, | Compound heterozygous | Hartnup disorder; autosomal recessive (234500) | 1-9/100.000 |
| 7 | M | Axonal polyneuropathy, spastic paraplegia | Adulthood | 2 | No | WES singleton | ***KIF5A*** | NM_004984.2 | c.967C>T, p.Arg323Trp | PVS1, PM1, PM2, PP2, PP3 | Heterozygous, de novo | Spastic paraplegia 10; autosomal dominant (604187) | <1/1.000.000 |
| 8 | M | Intracerebral hemorrhages, paralytic ileus, skin lesions | Neonatal | 19 | No | WES singleton | ***SGO1*** | NM_001012413.3 | c.67A>G, p.Lys23Glu | PP1, PP3, PP4, PP5, PM1 | Homozygous | Chronic atrial and intestinal dysrhythmia; autosomal recessive (616201) | <1/1.000.000 |
| 9 | F | ID, tremor, dyspraxia, hypogonadotropic hypogonadism | Neonatal | 19 | No | WES singleton | ***GALT*** | NM_000155.3 | c.563A>G, p.Gln188Arg | PVS1, PS3, PP5, PM1, PM2, PP2, PP3, PP4 | Homozygous | Galactosemia; autosomal recessive (230400) | 1/50.000 |
| 10 | M | Motor delay, autism, bilateral optic neuritis | Childhood | 15 | Yes | WES singleton | ***BTD*** | NM_001281723.1 | c.106G>A, p.Gly36Ser^(a)^;  c.1273T>C, p.Cys425Arg^(a)^ | PM1, PM2, PP2, BP4, PM3, PP4  PM1, PM2, PM5, PP2, PP3, PP4 | Compound heterozygous | Biotidinase deficiency; autosomal recessive (253260) | 1-9/100.000 |
| 11 | M | ID, epilepsy, tremor, dystonia, intracerebral cysts and calcifications | Neonatal | 44 | No | Targeted testing | ***SNORD118*** | NR_033294.1 | n.74G>A;  n.*10G>A | PM2, PM3, PP4, PP5  PM2, PM3, PP4, PP5 | Compound heterozygous | Leukoencephalopathy, brain calcifications, and cysts; autosomal recessive (614561) | <1/1.000.000 |
| 12 | M | Myalgia, hemolytic anemia | Adulthood | 7 | No | WES singleton | ***GATA1*** | NM_002049.3 | c.757C>T, p.Arg253Trp^(a)^ | PM1, PM2, PP3, PP4 | Hemizygous, maternally inherited | Anemia, X-linked, with/without neutropenia and/or platelet abnormalities (300835) | <1/1.000.000 |
| 13 | F | ID, cleft palate, dysmorphism, syndactyly, cardiomyopathy | Neonatal | 46 | No | CNVseq |  |  | **15 Mb deletion 4q34.1-q35.2** |  | Heterozygous, de novo | - | - |
| 14 | F | Polycystic kidney disease, cerebral white matter lesions, depression, anxiety, migraine | Neonatal | 52 | Yes | WES duo | ***PKD1***  ***COL4A1*** | NM_001009944.2  NM_001845.5 | c.3477C>G, p.Tyr1159Ter^(a)^  c.1144G>A, p.Gly382Ser^(a)^ | PVS1, PM2, PP3, PP4  PM1, PM2, PP2, PP3, PP1 | Heterozygous, maternally inherited | Polycystic kidney disease 1 (173900) Brain small vessel disease with or without ocular anomalies (175780) | 1-5/10.000  <1/1.000.000 |
| 15 | F | ID, epilepsy, tremor, facial dysmorphism, ventricular septum defect, polyposis | Neonatal | 23 | No | CNVseq |  |  | **29 Mb deletion 5q21.2-q31.1^(a)^** |  | Heterozygous, de novo | - | - |
| 16 | M | Cerebral white matter lesions, hallucinations, psychosis | Adulthood | 2 | No | WES singleton | ***COL4A1*** | NM_001845.5 | c.3715G>A, p.Gly1239Arg | PM1, PM2, PP2, PP3, PP5 | Heterozygous, de novo | Brain small vessel disease with or without ocular anomalies (175780) | <1/1.000.000 |
| 17 | F | Polycystic kidney disease | Adulthood | 14 | Yes | Targeted testing | ***PKD2*** | NM_000297.3 | c.710-2A>G, p.? | PVS1, PM2, PP3, PP5, PP4 | Heterozygous** | Polycystic kidney disease 2 (613095) | 1-5/10.000 |
| 18 | F | Torticollis, cervical vertebral fusions | Neonatal | 22 | No | WES singleton | ***RIPPLY2*** | NM_001009994.2 | c.240-4T>G, p.? | PM2, PP3, PP5, PP4 | Homozygous | Spondylocostal dysostosis 6 (616566) | Unknown |
| 19 | M | Neurofibromas, liposarcoma, prostate carcinoma, low-grade glioma | Adulthood | 3 | Yes | WES singleton | ***NF1***  ***TP53*** | NM_000267.3  NM_000546.5 | c.6905T>C, p.Leu2302Pro  c.845G>A, p.Arg282Gln | PM2, PP2, PP3, PP4  PM1, PM2, PM5, PP2, PP3 | Heterozygous** | Neurofibromatosis, type 1 (162200) Li-Fraumeni syndrome (151623) | 1-5/10.000  Unknown |
| 20 | M | Congenital hearing loss | Neonatal | 33 | Yes | Targeted testing | ***GJB2*** | NM_004004.5 | c.35del, p.Gly12ValfsTer2;  c.-23+1G>A, p.? | PVS1, PS3, PP5, PP3  PVS1, PP5, PM2, BP4 | Compound heterozygous | Deafness, autosomal recessive 1A (220290) | Unknown |
| 21 | F | Small stature, cardiomyopathy, hearing loss, cervical vertebral fusions, facial dysmorphism | Childhood | 14 | No | WES trio | ***MAP3K7*** | NM_145331.2 | c.571G>A, p.Gly191Arg^(a)^ | PM1, PM2, PP2, PP3, PS2, PS3 | Heterozygous, de novo | Cardiospondylocarpofacial syndrome (157800) | <1/1.000.000 |
| 22 | M | ID, epilepsy, autism, aggression, auto mutilation | Neonatal | 19 | No | WES trio | ***OPHN1*** | NM_002547.2 | c.1226G>A, p.Arg409His^(a)^ | PM1, PM2, PM5, PP3 | Hemizygous, maternally inherited (mosaic mother) | Mental retardation, X-linked, with cerebellar hypoplasia and distinctive facial appearance (300486) | <1.000.000 |
| 23 | F | Lipodystrophy, muscular hypertrophy, diabetes mellitus, dyslipidemia, hepatic steatosis | Adolescence | 19 | Yes | WES* trio | ***PLAAT3*** | NM_001128203.1 | c.16-4823_118+167del, p.Pro6ValfsTer15^(b)^ | PVS1, PM2, PP3, PS3, PP1 | Homozygous | ? Familial partial lipodystrophy, type 8, autosomal recessive | Unknown |
| 24 | M | Leiomyomata, renal failure | Adulthood | 5 | No | CNVseq | ***FH*** |  | **2.9 Mb deletion 1q43^(a)^** |  | Heterozygous, de novo | Hereditary leiomyomatosis and renal cell cancer (150800) | Unknown |
| 25 | M | Spinocerebellar ataxia, cerebellar atrophy | Adulthood | 48 | Yes | WES* singleton | ***ITPR1*** |  | **240 kb deletion 3p26.1** |  | Heterozygous, paternally inherited | Spinocerebellar ataxia 15 (606658) | <1/1.000.000 |
| 26 | F | Non compaction cardiomyopathy, polycystic kidney disease | Adulthood | 3 | Yes | WES singleton | ***TTN***  ***PKD1*** | NM_001267550.2  NM_001009944.2 | c.49793G>A, p.Trp16598Ter^(a)^  c.9241T>C, p.Cys3081Arg^(a)^ | PVS1, PM2, PP3, PP4  PM2, PP2, PP3, PP4, PP1 | Heterozygous** | Cardiomyopathy, familial hypertrophic, 9 (613765)/Cardiomyopathy, dilated, 1G (604145) Polycystic kidney disease 1 (173900) | Unknown  1-5/10.000 |
| 27 | F | Cerebral white matter lesions | Adulthood | 1 | Yes | WES singleton | ***HTRA1*** | NM_002775.4 | c.511T>C, p.Phe171Leu^(a)^ | PM2, PP2, PP3, PP1 | Heterozygous, paternally inherited | Cerebral arteriopathy, autosomal dominant, with subcortical infarcts and leukoencephalopathy, type 2 (616779) | <1/1.000.000 |
| 28 | M | Ichthyosis | Childhood | 42 | No | WES singleton | ***FLG*** | NM_002016.1 | c.1501C>T, p.Arg501Ter  c.2282_2285del, p.Ser761Cysfs*36 | PVS1, PP5  PVS1, PP5 | Compound heterozygous | Ichthyosis vulgaris (146700) | 7/100 |
| 29 | F | Liver cysts | Adulthood | 2 | Yes | WES singleton | ***GANAB*** | NM_198334.2 | c.38G>A, p.Arg13Gln | PVS1, PM1, PM2, PP3, PP5, PP4, PP1 | Heterozygous, maternally inherited | Polycystic kidney disease 3 (600666) | 1-5/10.000 |
| 30 | M | Recurrent stroke, cerebral white matter lesions | Adulthood | 3 | Yes | WES singleton | ***HTRA1*** | NM_002775.4 | c.847G>A, p.Gly283Arg | PM1, PM2, PP5, PP2, PP3 | Heterozygous** | Cerebral arteriopathy, autosomal dominant, with subcortical infarcts and leukoencephalopathy, type 2 (616779) | <1/1.000.000 |
| 31 | F | ID, microphthalmia, facial dysmorphism | Neonatal | 43 | No | WES duo | ***KAT6B*** | NM_012330.3 | c.5821del, p.Ser1941HisfsTer10^(a)^ | PVS1, PM2, PP3, PS2 | Heterozygous, de novo | SBBYSS syndrome (603736) | <1/1.000.000 |
| 32 | M | Kidney cysts, renal failure | Adulthood | 18 | Yes | WES singleton | ***HNF1B*** | NM_000458.3 | c.1060del, p.Gln354SerfsTer22^(a)^ | PVS1, PM2, PP3, PP1 | Heterozygous** | Renal cysts and diabetes syndrome (137920) | 1-9/1.000.000 |
| 33 | F | Episodic fever, urticaria | Adulthood | 9 | No | WES singleton | ***NLRP3*** | NM_001243133.1 | c.592G>A, p.Val198Met | PS4, PP5, PS3, BS1 | Heterozygous** | Muckle-Wells syndrome (191900) | Unknown |
| 34 | F | Episodic fever, urticaria | Adulthood | 4 | *NA* | WES singleton | ***NLRP3*** | NM_001243133.1 | c.592G>A, p.Val198Met | PS4, PP5, PS3, BS1 | Heterozygous** | Muckle-Wells syndrome (191900) | Unknown |
| 35 | M | Pheochromocytoma | Adolescence | 25 | Yes | WES singleton | ***SDHA*** | NM_004168.3 | c.1808A>G, p.Glu603Gly | PM2, PP3, PP5, PP4, PP1 | Heterozygous, maternally inherited | Paragangliomas 5 (614165) | 1-9/1.000.000 |
| 36 | F | Bilateral carotid body tumor | Adulthood | 18 | Yes | Targeted testing | ***SDHD*** | NM_003002.3 | c.170-1G>T, p.? | PVS1, PM2, PP3, PP5, PP4 | Heterozygous** | Paragangliomas 1, with or without deafness (168000) | 1-9/1.000.000 |
| 37 | F | Recurrent carotid body tumor | Adulthood | 33 | Yes | WES singleton | ***SDHD*** | NM_003002.3 | c.242C>T, p.Pro81Leu | PP5, PM1, PM2, PP2, PP3, PP4 | Heterozygous** | Paragangliomas 1, with or without deafness (168000) | 1-9/1.000.000 |
| 38 | M | Hematuria, proteinuria, renal failure | Adolescence | 40 | Yes | Targeted testing | ***COL4A3*** | NM_000091.4 | c.4033C>G, p.Arg1345Gly^(a)^ | PM1, PM2, PP2, BP4, PP4 | Heterozygous** | Alport syndrome 3, autosomal dominant (104200) | Unknown |
| 39 | M | Hematuria | *NA* | *NA* | Yes | WES singleton | ***COL4A4*** | NM_000092.4 | c.3022G>A, p.Gly1008Arg | PM1, PM2, PP2, PP3, PP4 | Heterozygous** | Hematuria, familial benign (141200) | Unknown |
| 40 | M | Cerebral white matter lesions, hearing loss | Adulthood | 1 | Yes | WES trio | ***HTRA1*** | NM_002775.4 | c.1009G>A, p.Gly337Ser^(a)^ | PM1, PM2, PP2, PP3, PP1 | Heterozygous, maternally inherited | Cerebral arteriopathy, autosomal dominant, with subcortical infarcts and leukoencephalopathy, type 2 (616779) | <1/1.000.000 |
| 41 | F | Recurrent fever, skin rash | Adulthood | 4 | *NA* | WES singleton | ***NLRP3*** | NM_001243133.1 | c.592G>A, p.Val198Met | PS4, PP5, PS3, BS1 | Heterozygous** | Muckle-Wells syndrome (191900) | Unknown |
| 42 | F | Cerebral white matter lesions, pain, chilblains, dermatitis | Adulthood | 5 | *NA* | WES singleton | ***SAMHD1*** | NM_015474.3 | c.901G>C, p.Val301Leu^(a)^ | PM1, PM2, PP2, PP3, PP4 | Heterozygous** | Chilblain lupus 2 (614415) | <1/1.000.000 |
| 43 | F | Epilepsy, parasagittal occipital polymicrogyria | Adolescence | 7 | No | CNVseq |  |  | **3 Mb duplication 22q11.2** |  | Heterozygous** | 22q11.2 microduplication syndrome (608363) | Unknown |
| 44 | F | Arthralgia, urticaria, aphthous ulcers, episodic fever, hearing loss | Childhood | 32 | Yes | WES singleton | ***NLRP12*** | NM_144687.3 | c.430A>G, p.Asn144Asp^(a)^ | PP1, PP4, PS3 | Heterozygous, paternally inherited | Familial cold autoinflammatory syndrome 2 (611762) | <1/1.000.000 |
| 45 | F | Cerebral white matter lesions, epilepsy, mild ID | Childhood | 32 | Yes | WES trio | ***COL4A2*** | NM_001846.2 | c.2588G>A, p.Gly863Asp^(a)^ | PVS1, PM1, PM2, PP3, PP4, BP1 | Heterozygous, maternally inherited | Brain small vessel disease 2 (614483) | Unknown |
| 46 | M | ID, psychiatric disorders, myopathy, axonal polyneuropathy, dysarthria, facial dysmorphism | Childhood | 41 | No | WES singleton | ***PHIP*** | NM_017934.6 | c.41-1G>A, p.?^(a)^ | PVS1, PM2, PS2 | Heterozygous, de novo | Chung-Jansen syndrome (617991) | Unknown |
| 47 | F | Bilateral avascular necrosis hip, avascular necrosis shoulder | Adulthood | 5 | No | WES singleton | ***COL2A1*** | NM_001844.4 | c.4436T>C, p.Val1479Ala^(a)^ | PM1, PM2, PP2, PP3, PP4 | Heterozygous** | Avascular necrosis of the femoral head (608805) | Unknown |
| 48 | M | Muscle weakness, myalgia axial musculature, failed back surgery, cardiomyopathy | Adulthood | 10 | No | WES duo | ***SELENON*** | NM_020451.2 | c.997_1000del, p.Val333ProfsTer?  c.1339G>A, p.Val447Met^(a)^ | PVS1, PM2, PP5, PP3, PP4  PM2, PP2, PP3, PP4, PM3 | Compound heterozygous | Muscular dystrophy, rigid spine, 1 (602771) | Unknown |
| 49 | F | Focal dermal hypoplasia, dystrophic nails, enamel hypoplasia, scoliosis, syndactyly 2^nd^/3^rd^ toe, papillomas | Neonatal | 40 | No | Tareted testing | ***PORCN*** | NM_203473.2 | c.484dup, p.Val162GlyfsTer63^(a)^ | PVS1, PM2, PP3, PP4 | Mosaic | Focal dermal hypoplasia (300651) | <1/1.000.000 |
| 50 | M | Demyelinating polyneuropathy, pes cavus | *NA* | *NA* | Yes | Targeted testing | ***PMP22*** |  | **17p12 microduplication** |  | Heterozygous, maternally inherited | Charcot-Marie-Tooth Neuropathy type 1A (118220) | 1-5/10.000 |
| 51 | F | Cerebral white matter lesions, depression, hallucinations, motor problems, dysarthria, epilepsy | Adulthood | 1 | No | WES singleton | ***ACMSD*** | NM_138326.2 | c.547C>T, p.Arg183Ter^(b)^ | PVS1, PP3 | Homozygous | ? ACMSD-deficiency | - |
| 52 | M | Intellectual disability, periventricular leukomalacia, cerebral calcifications | Neonatal | 19 | No | Targeted analysis | ***RNU7-1*** | NR_023317.1 | n.40_47del;  n.27dup^(a)^ | PVS1, PM3, PP4, PP5 PVS1, PM3, PP4 | Compound heterozygous | Aicardi-Goutieres syndrome 9 (619487) | Unknown |
| 53 | V | External ophtalmoplegia, myopathy with ragged red fibers on biopsy | Adulthood | 22 | No | mtDNA sequencing (muscle tissue) |  | NC_012920.1 | **m.12113_14421del (60% heteroplasmy)** |  | Mitochondrial DNA | Large-scale single deletion of mitochondrial DNA: Progressive external ophthalmoplegia | Unknown |

*Making use of an algorithm (ExomeDepth) to detect copy number variants in WES data. All indels were confirmed by CNV sequencing

**Parents not tested

^(a)^New mutation in known disease gene

^(b)^New disease gene

*NA*: not available
